# Intracellular Acidification Suppresses Synaptic Vesicle Mobilization in the Motor Nerve Terminals

**DOI:** 10.32607/actanaturae.11054

**Published:** 2020

**Authors:** A. L. Zefirov, R. D. Mukhametzyanov, A. V. Zakharov, K. A. Mukhutdinova, U. G. Odnoshivkina, A. M. Petrov

**Affiliations:** Kazan State Medical University, Department of Normal Physiology, Kazan, 420012 Russia; Institute of Neuroscience, Kazan State Medical University, Kazan, 420012 Russia; Kazan Federal University, Kazan, 420008 Russia; Laboratory of Biophysics of Synaptic Processes, Kazan Institute of Biochemistry and Biophysics, Federal Research Center “Kazan Scientific Center of RAS”, Kazan, 420111 Russia

**Keywords:** exocytosis, synaptic vesicle translocation, neurotransmission, acidification, neuromuscular junction

## Abstract

Intracellular protons play a special role in the regulation of presynaptic processes, since the functioning of synaptic vesicles and endosomes depends on their acidification by the H+-pump. Furthermore, transient acidification of the intraterminal space occurs during synaptic activity. Using microelectrode recording of postsynaptic responses (an indicator of neurotransmitter release) and exo-endocytic marker FM1-43, we studied the effects of intracellular acidification with propionate on the presynaptic events underlying neurotransmitter release. Cytoplasmic acidification led to a marked decrease in neurotransmitter release during the first minute of a 20-Hz stimulation in the neuromuscular junctions of mouse diaphragm and frog cutaneous pectoris muscle. This was accompanied by a reduction in the FM1-43 loss during synaptic vesicle exocytosis in response to the stimulation. Estimation of the endocytic uptake of FM1-43 showed no disruption in synaptic vesicle endocytosis. Acidification completely prevented the action of the cell-membrane permeable compound 24-hydroxycholesterol, which can enhance synaptic vesicle mobilization. Thus, the obtained results suggest that an increase in [H+]in negatively regulates neurotransmission due to the suppression of synaptic vesicle delivery to the sites of exocytosis at high activity. This mechanism can be a part of the negative feedback loop in regulating neurotransmitter release.

## INTRODUCTION

Synaptic transmission is based on neurotransmitter release from synaptic vesicles (SVs) via exocytosis in response to the arrival of an action potential from an axon to the nerve ending (NE). This mechanism is universal and depends on the transport (mobilization) of SVs to the sites of exocytosis, the so-called active zones (AZs), where the proteins involved in exocytosis and voltage-gated Ca^2+^ channels are concentrated [1]. In turn, the mobilization depends on the number of available SVs located near the AZs and the supply of SVs newly formed via endocytosis immediately after exocytosis. Under conditions of continuous rhythmic or moderate-frequency activity, the mobilization rate is responsible for the level of neurotransmitter release and, consequently, the reliability of neurotransmission [2, 3].

The fundamental mechanisms regulating SV mobilization remain insufficiently understood. The cytoskeleton, motor proteins, and small GTPases were shown to play an important role in the regulation of SV transport [4]. However, the significance of such an important factor as the cytoplasmic pH has not been clarified. Meanwhile, it is known that changes in pH_in_ related to proton pumping and the function of vesicular neurotransmitter transporters occur in NEs during synaptic activity. Neurotransmitter transporters exchanging a neurotransmitter molecule for proton(s) are incorporated into the presynaptic membrane after exocytosis of SVs, while maintaining their functional activity [5, 6]. The Ca^2+^/H^+^ exchange by Ca^2+^ ATPase of the presynaptic plasma membrane can also take part in cytoplasmic acidification in response to increased [Ca^2+^]_in_ during depolarization, while the Na^+^/H^+^ exchanger is involved in pHin restoration in NEs [7]. Intense stimulation was shown to decrease pH of the NE cytosol in the neuromuscular junctions of a fruit fly, mouse, and rat [5, 6, 7]. However, it remains unclear what effect intracellular acidosis caused by synaptic activity has on presynaptic processes.

The early studies showed that an abrupt drop in pH of cells can inhibit clathrin-mediated endocytosis [8]. This can be a result of impaired clathrin coat assembly, dysfunction of adapter proteins, or decreased synthesis of phosphatidylinositol-4,5- bisphosphates [9, 10]. However, the same cannot be extrapolated to the synaptic machinery, since endocytosis in the synapse is highly specific and requires a specific set of proteins to become involved. In addition, several types of endocytosis, including clathrin-independent ones, coexist in the synapse [11]. For example, a carbonic anhydrase inhibitor switches the type of endocytosis in the neuromuscular junctions of mice to a clathrin-independent one by lowering the cytosolic pH [12].

In general, it remains unclear how a reduced cytoplasmic pH can affect neurotransmitter release and the mobilization of SVs during continuous activity. In the present study, using electrophysiological detection of neurotransmitter release and a fluorescence-based method for tracking the exocytosis and endocytosis, we were able to demonstrate for the first time that intracellular acidification can significantly inhibit SV mobilization in the neuromuscular junctions of cold- and warm-blooded animals. We suggest that this phenomenon may be a new physiological mechanism regulating the SV transport during synaptic activity.

## EXPERIMENTAL

Our experiments were carried out using isolated neuromuscular preparations from the diaphragm muscle of white laboratory mice and the cutaneous pectoris muscle of frogs (*Rana ridibunda*) in autumn and winter, in compliance with the Guide for the Care and Use of Laboratory Animals. The experiment protocol complied with European Directive 2010/63/EU on the protection of animals used for scientific purposes and was approved by the Ethics Committee of the Kazan Medical University.

**Solutions and reagents**

The muscle was attached to the bottom of a 5-mL chamber under continuous perfusion. An oxygenated Krebs solution of the following composition was used in the experiments performed on the mouse muscle: 144.0 mM NaCl, 5.0 mM KCl, 0.1 mM MgCl_2_, 2.0 mM CaCl_2_, 1.0 mM NaH_2_PO_4_, 2.4 mM NaHCO_3_, and 11.0 mM glucose. Ringer’s solution (115.0 mM NaCl, 2.5 mM KCl, 1.8 mM CaCl_2_, and 2.4 mM NaHCO_3_) was used in the experiments performed on the frog muscle. The pH of the solutions was maintained at 7.3–7.4 at a temperature of 20°C. D-tubocurarine (2–5 μM) was used to avoid muscle contraction. Modified Krebs and Ringer solutions, with sodium chloride partially replaced with sodium propionate (namely, 72 mM), were used to induce intracellular acidification. The resulting concentration of sodium propionate in the modified solutions was 72 mM; pH and osmolality were maintained identical to those of normal saline. Reagents procured from Sigma-Aldrich (USA) were used. The experiments were started after perfusion of the preparations with propionate solutions for 45–50 min; 24-hydroxycholesterol (0.4 μM) was applied for 15 min.

**Electrophysiology**

End-plate potentials (EPPs) were recorded intracellularly using glass microelectrodes (tip diameter < 1 µm; resistance, 5–20 mΩ) filled with 3 M KCl. The amplifier Model 1600 (A-M Systems, USA) and an LA-2USB analog-to-digital converter were used to amplify and record EPPs under the control of the Elph software [13]. The motor nerve was stimulated by rectangular 0.1–0.2 ms pulses at a frequency of 20 Hz for 3 min (Model 2100 Stimulator, A-M Systems, USA). The stimulation frequency was then reduced to 0.3 Hz, and the recovery of the EPP amplitude was recorded [14, 15].

The quantum content of EPPs was calculated using the modified method of variations described earlier in details [3]. For this purpose, the area of each EPP in the series was determined. Further, the region on the diagram showing the reduction in the EPP area during high-frequency stimulation in which the average EPP area remained practically unchanged was identified (the plateau phase, which usually lasts the first 10–30 s). The quantum value (i.e., the average area of EPPs produced by a single neurotransmitter quantum) can be calculated from the fluctuations in the EPP area within this region (*q*): *q *= σ^2^/ < *V*>, where σ is the standard deviation of the EPP area, and < *V*> is the average EPP area in this region. Next, one can determine the quantum content of each EPP in the series: *mi *= *V_i_*/*q*, where *m_i_*is the quantum content of the *i*_th_ EPP, and *V_i_*is the area of the *i*^th^ EPP.

**Fluorescence microscopy**

Fluorescence was observed using an Olympus BX-51WI microscope. An Olympus UPLANSapo lens (60× magnification) and a LumPlanPF lens (100× magnification) were used. Images were recorded using an Olympus DP71 camera and processed using the CellSens software (Olympus). The ImagePro (Media Cybernetics) software was employed for the fluorescence analysis.

A FM1-43 dye (5 μM) was used to assess the endo-/exocytosis of SVs. FM1-43 binds reversibly to the presynaptic membrane and is loaded into the newly formed SVs during endocytosis. Fluorescent spots appear as NEs are loaded with the dye, indicating that FM1-43 containing SVs are clustered in the AZ regions [16, 17]. To assess the endocytosis of SVs, FM1-43 was kept present during the stimulation and for 10 min afterwards to ensure that endocytosis caused by exocytosis stimulation had ended by that time. Next, the preparation was washed for 40 min in saline containing the ADVASEP-7 reagent (5 μM), which promotes the dissociation of FM1-43 from surface membranes [18]. The SVs that are formed during endocytosis and retain the FM1-43 dye start losing the dye in a new round of exocytosis. To assess the dynamics of the exocytosis, the preparations preloaded with FM1-43 (20-Hz stimulation for 3 min) were re-stimulated (at 20 Hz frequency, 10–20 min) and the decrease in the fluorescence intensity due to dye unloading was analyzed [19]. The properties of the FM1-43 marker are independent of pH in a pH range of 5–9 [20]; 0.4 µM 24-HC also does not affect the fluorescence of FM1-43 [21, 22].

The fluorescence of FM1-43 was detected using an excitation filter (480/10 nm), a dichroic mirror (505 nm), and an emission filter (535/40 nm). Fluorescence was evaluated as the average pixel intensity in the region of interest after subtracting the background fluorescence. When determining the rate of dye loss during unloading, the initial fluorescence of NE before the stimulation had started was assumed to be equal to 1.0.

The BCECF-AM ratiometric fluorescent probe (Molecular Probes, USA) was used as an indicator of cytoplasmic pH. The muscle specimens were incubated with the dye at a concentration of 5 μM for 15 min and then perfused for 30 min to reduce the background fluorescence. The dye-loaded synaptic contacts were exposed to intermittent light (1 s, 505/10 and 450/10 nm). Fluorescence was detected in the synaptic region using a 530-nm broadband emission filter. The *I*^505^/*I*^450^ fluorescence ratio upon excitation by two wavelengths was used to estimate the intracellular pH. The decreased *I*^505^/*I*^450^ ratio indicates that the intracellular pH had dropped. At the end of each experiment, the muscle specimens were perfused with phosphate buffer (138 mM NaCl, 2.7 mM KCl, 10 mM Na_2_HPO_4_, and 1.8 mM KH_2_PO_4_) containing 10 μM nigericin to equalize the extra- and intracellular pH. The *I*^505^/*I*^450^ ratio was evaluated when the preparation was exposed to buffer with different pH values (7.4–7.1) for calibration [5].

**Statistical analysis**

The results are presented as a mean ± standard error; *n *is the number of independent experiments performed on individual animals (indicated in the figure legends). The Mann–Whitney U test was used to compare two independent samples. Differences at *P * < 0.05 were considered statistically significant.

## RESULTS

**Monitoring of intracellular acidification**

**Fig. 1:**
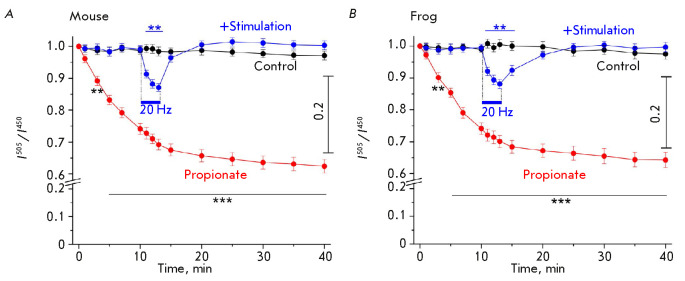
Monitoring of cytosolic pH in the synaptic regions. The ratio between BCECF dye fluorescence intensities upon 505 and 450 nm excitation (*I*^505^/*I*^450^) is an indicator of pH and decreases in response to pH_in_ reduction. (*A*), (*B*) – the measurements of the *I*^505^/*I*^450^ ratio in the synaptic regions of mouse (*A*) and frog (*B*) preparations at rest (control), exposed to 20-Hz stimulation for 3 min (shown in blue), and in the presence of propionate (72 μM; shown in red). Y axis: the *I*^505^/*I*^450^ ratio at the initial instant is set at 1.0; *n *= 7 for each curve. The right scale shows the decrease in the *I*^505^/*I*^450^ ratio in response to pH drop by 0.2 pH units. Data are presented as a mean ± SEM. **P  < 0.01, ***P  < 0.001 is the statistical significance of the differences between the curves

Anions of weak acids are known to acidify the intracellular environment. Propionate is widely used to mimic intracellular acidosis [23]. The undissociated form of propionic acid enters the cytoplasm and dissociates, thus decreasing the pH_in_. Indeed, measurements of cytoplasmic pH using BCECF showed that application of propionate reduced the *I*^505^/*I*^450^ ratio. This was an indication that pH in the synaptic zone of the mouse and frog preparations decreased (*Fig. 1 A, B*). After 40 min of exposure to propionate, the *I*^505^/*I*^450^ ratio fell to its steady-state level, being ~ 60–65% of the baseline (by ~0.25 pH units). This is comparable to the previously estimated change in pH_in_ in rat synapses in the presence of propionate [5]. In the control, the *I*^505^/*I*^450^ ratio remained unchanged for 40 min (*Fig. 1 A, B*), indicating that pH_in_ is stable at rest. Stimulation (at a frequency of 20 Hz) transiently reduced the *I*^505^/*I*^450^ ratio in the synaptic region (*Fig. 1 A, B*). This is consistent with the concept of intracellular acidification of NEs taking place during synaptic activity; once there remains no activity, pH_in_ is slowly restored [5, 6, 7].

**Dynamics of neurotransmitter release and the effect of intracellular acidification**

**Fig. 2:**
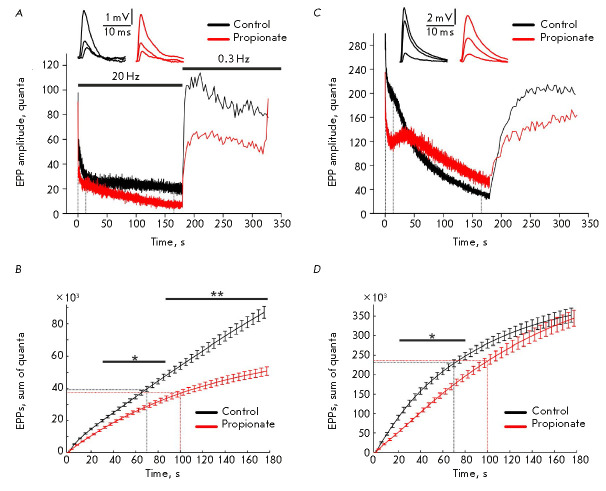
The kinetics of neurotransmitter release at a 20-Hz stimulation. (*A*), (*C*) – Stimulation-induced changes in the quantum content of EPPs in the neuromuscular junctions in a mouse (*A*) and a frog (*C*) under control conditions and upon intracellular acidification with propionate. The recovery of the quantum content of EPPs after the 20-Hz stimulation is also shown. The native EPPs recorded at the moment of stimulation marked with dashed lines on the graph are shown at the top. Averaged curves are presented; *n *= 5. (*B*), (*D*) – The cumulative curves of the quantum content of EPPs in a 20-Hz stimulation in the neuromuscular junctions of a mouse (*B*) and a frog (*D*). Data are presented as a mean ± SEM. *P  < 0.05, **P < 0.01 is the statistical significance of the differences between the curves; *n *= 5. The dashed lines denote the time points (70 and 100 s) at which the same number of quanta was released in the control and in the presence of propionate

Long-term synaptic activity is maintained due to SV mobilization from the recycling and reserve pools to the AZ, followed by subsequent neurotransmitter release [1, 3]. In the control, the quantum content of EPPs during stimulation of the mouse phrenic nerve by electric pulses at a frequency of 20 Hz dropped rapidly during the first 5–10 pulses, to 20–25% of the baseline (155 ± 20 quanta). The quantum content was then stabilized and slowly decreased down to 10–15% of the baseline by the 3^rd^ min of stimulation. Once the stimulation had been completed, the quantum content of EPPs was quickly restored to 50% of the baseline (6 ± 2 s, *Fig. 2A*). These changes in EPPs in response to 20-Hz stimulation and the rapid recovery of secretion are consistent with the view that neurotransmitter release in mouse motor NEs during 20-Hz stimulation is first maintained by the vesicles constituting the readily releasable pool and then by the vesicles of the recycling pool [2, 3]. SVs of the recycling pool are rapidly recovered by endocytosis and then re-used in the neurotransmitter release.

Application of propionate caused no statistically significant changes in the quantum content of the first EPP (128 ± 17 quanta, *P* < 0.05). However, it significantly accelerated the rundown of EPPs in the neuromuscular junctions of mice (*Fig. 2A*). The quantum content eventually decreased to 3–5% of the baseline by the 3^rd^ min of 20-Hz stimulation. Recovery of the quantum content after the stimulation had ended was slower (up to 50% from the baseline within 13 ± 3 s) than that in the control. In order to quantify the neurotransmitter release, the cumulative curves were plotted by summing up the quantum contents of each EPP during 20-Hz stimulation for 3 min and the total number of quanta released from NEs was determined. A total of (90 ± 3.9) × 10^3^ quanta were released in the control during the 3-min stimulation. This value was significantly lower in the presence of propionate (*P* < 0.01): (51 ± 2.8) × 10^3^ quanta (*Fig. 2B*). Therefore, intracellular acidification of NEs in mice significantly inhibits neurotransmission during high synaptic activity by weakening the release of the neurotransmitter from the recycling pool of SVs.

Stimulation of the motor nerve of the frog cutaneous pectoris muscle at a frequency of 20 Hz was first accompanied by a reduction (during 30–40 stimuli) in the quantum content of EPPs to approximately 80% of the baseline (272 ± 30 quanta). The quantum content was stabilized by 3–5 s (the plateau) and then gradually decreased to 10–15% of the baseline value by the 3^rd^ min of stimulation (*Fig. 2C*). It took 18 ± 3 s for the quantum content after the 20-Hz stimulation had completed to recover, reaching 50% of the baseline. These dynamics indicate that not only the readily releasable and the recycling pools are involved in neurotransmitter release, but the reserve pool as well [2, 3, 24].

The quantum content of the first EPP in the frog cutaneous pectoris muscle exposed to propionate had no difference compared to the control and was equal to 227 ± 35 quanta. The initial EPP inhibition was more pronounced, while the plateau phase was longer. The recovery of the quantum content after the 20-Hz stimulation was slower than that in the control (up to 50% within 21 ± 3 s) (*Fig. 2C*). By comparing the cumulative curves of the quantum contents of EPPs, one can see that neurotransmitter release decreases in the presence of propionate, being pronounced during the first minute of the 20-Hz stimulation (*Fig. 2D*). By the 3^rd^ min of stimulation, the neurotransmitter release reached control values and amounted to (347 ± 13) × 10^3^ quanta (vs. (355 ± 17) × 10^3^ quanta in the control). Thus, intracellular acidification inhibits neurotransmitter release in frog NEs during the period when secretion is mediated by the recycling pool SVs.

**Endo- and exocytosis during intracellular acidification**

**Fig. 3:**
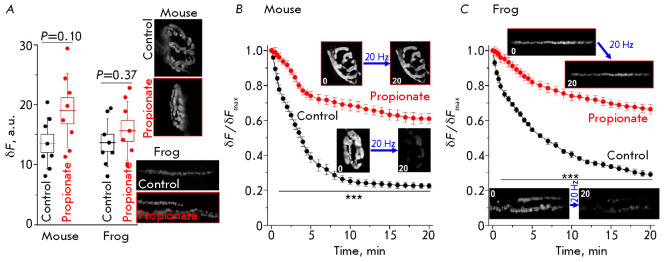
Endocytosis and exocytosis in response to the 20-Hz stimulation. (*A*) – FM1-43 uptake by endocytosis in motor NEs in the control and in the presence of propionate. Boxes and whiskers show SEM and SD, respectively. Right, representative fluorescence images after the background was subtracted, *n *= 8 for each group. Y axis: fluorescence in arbitrary units (a.u.) after background subtraction. (*B*), (*C*) – FM1-43 unloading due to exocytosis during the 20-Hz stimulation in the control and in the presence of propionate in the neuromuscular junctions of a mouse (*B*) and a frog (*C*). *n *= 8 for each curve. The images illustrate a decrease in the NE fluorescence after 20 min of stimulation. Data are presented as a mean ± SEM. ***P  < 0.001 – statistical significance of the differences between the curves. Y axis: normalized fluorescence, where 1.0 is the fluorescence before the onset of stimulation

*Endocytosis*. Considering that acidification in NEs inhibited the neurotransmitter release, which was dependent on the recycling pool of SVs, endocytosis dysfunction is quite possible. In order to test this hypothesis, we evaluated the endocytic uptake of FM1-43 in NEs. SV endocytosis follows exocytosis and is carried out at a 1 : 1 ratio. Therefore, we selected the duration of 20-Hz stimulation, at which the same neurotransmitter release level was observed in both the control and experimental series (and, therefore, the same number of SVs underwent exocytosis). An analysis of the cumulative curves of neurotransmitter release showed that approximately the same number of neurotransmitter quanta was released during a 70-s stimulation in the control and a 100-s stimulation in the presence of propionate (*Fig. 2 B, D*). If endocytosis is not disrupted, a similar level of FM1-43 loading can be expected under the chosen conditions. Indeed, the fluorescence intensities of mouse and frog NEs in the presence of sodium propionate did not significantly differ from those in the control (*Fig. 3A*).

*The exocytosis kinetics*. The dynamics of SV exocytosis during long-term 20-Hz stimulation was assessed by measuring the unloading of the FM1-43 dye from NEs. In the synapses of the control mice, fluorescence gradually decreased to 25–30% within 10 min of stimulation and then changed slowly (*Fig. 3B*). FM1-43 unloading was significantly impeded upon cytoplasmic acidification, and the fluorescence intensity decreased only to ~70% of the baseline by the 10^th^ min of stimulation (*Fig. 3B*). Therefore, propionate inhibits the involvement in exocytosis of SVs, which maintain neurotransmission in mice at 20-Hz stimulation.

In control, the decrease in the fluorescence intensity occurred in two phases in the frog NEs: a rapid drop during the first 2 min (up to 70% of the baseline), followed by a slower decrease (*Fig. 3C*). By the 20th min of stimulation, the fluorescence intensity had dropped to 25–30%. FM1-43 unloading was inhibited upon intracellular acidification. The rate of FM1-43 unloading decreased most significantly within the first 2 min of stimulation (the fluorescence intensity declined only to ~95% of the baseline). By the 20th min of the 20-Hz stimulation, the fluorescence intensity had decreased to ~70% of the baseline (*Fig. 3C*). Therefore, propionate markedly inhibits the involvement in exocytosis of the recycling pool SVs, which maintain neurotransmission during the first several minutes of the 20-Hz stimulation of the motor nerve in frog NEs.

**Intracellular acidification and the effect of 24-hydroxycholesterol on the changes in exocytosis**

**Fig. 4:**
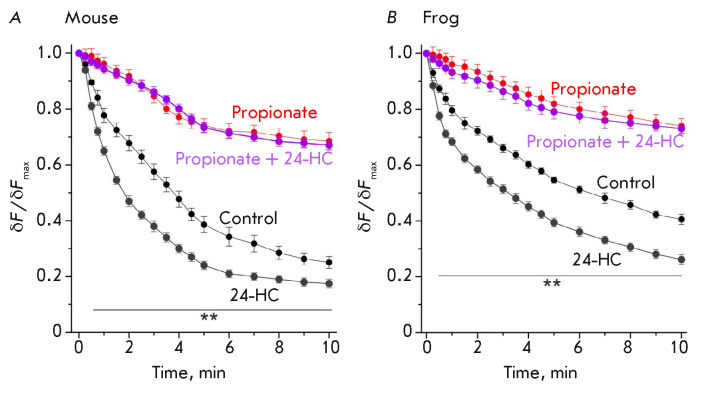
The influence of cytoplasmic acidification on the effect of 24-hydroxycholesterol (24-HC) on exocytosis in a 20- Hz stimulation. (*A*), (*B*) – changes in the kinetics of FM1-43 unloading due to the administration of 24-HC in the control and in the presence of propionate in the NEs of a mouse (*A*) and a frog (*B*). The control and propionate curves (from *Fig. 3 B, C*) are also shown. Data are presented as a mean ± SEM. **P  < 0.01 – statistical significance of the differences between the control and the effect of 24-HC. Y axis: normalized fluorescence, where 1.0 is the fluorescence prior to the onset of stimulation

We have previously shown that 24-hydroxycholesterol can enhance SV mobilization in neuromuscular junctions upon 20-Hz stimulation [21]. Exposure to 24-hydroxycholesterol (0.4 μM) accelerated FM1-43 unloading upon the 20-Hz stimulation (*Fig. 4 A, B*). A similar effect was observed in the NEs of mice and frogs. In the presence of propionate, 24-hydroxycholesterol completely lost its ability to accelerate the rate of FM1-43 release during exocytosis (*Fig. 4 A, B*). Hence, intracellular acidification rendered acceleration of SV mobilization upon the 20-Hz stimulation impossible.

## DISCUSSION

Numerous regulatory circuits acting on exocytosis, mobilization, and endocytosis of SVs establish the proper level of neurotransmitter release during synaptic activity. In the present study, we obtained data on the suppression of SV mobilization upon intracellular acidification for the first time. Furthermore, this phenomenon was observed in the NEs of both mice and frogs, thus indicating that the general mechanisms of intracellular acidosis action are identical.

Propionate efficiently reduced the intracellular pH by ~ 0.25 pH units, which is twofold higher than the degree of acidification caused by motor nerve stimulation with 20-Hz pulses. An analysis of postsynaptic responses showed that propionate did not significantly change the quantum content in response to the first stimulus, while accelerating the depression of neurotransmitter release in response to the 20-Hz stimulation. Under these conditions, the neurotransmitter release depends on the delivery of SVs to the AZ. In mouse synapses, the effect of propionate was clearly observable throughout the entire stimulation. On the contrary, the effect was observed only during the first minute of stimulation in frog synapses. These features of the effect of propionate are probably related to the specific involvement of SV pools in neurotransmission upon a 20-Hz stimulation. In particular, the recycling pool sustains long-term neurotransmitter release in mouse motor NEs upon a 20-Hz stimulation. Meanwhile, in frog motor NEs, this pool maintains the release mainly during the first minute of the stimulation, after which the reserve pool SVs become involved in neurotransmission. Hence, propionate seems to inhibit the involvement of the recycling pool SVs in the release. This selectivity of intracellular acidosis is consistent with the concept that there are independent pathways that regulate the recycling and reserve pools [15, 19, 25, 26, 27]. Moreover, the rate of propionate-mediated inhibition of neurotransmitter release in the frog NEs decreased after 60 s. As a result, the number of released transmitter quanta differed little from that in the control after stimulation for 3 min. Inhibition of the recruitment of SVs from the recycling pool seems to contribute to the release of the neurotransmitter from SVs belonging to the reserve pool.

The involvement of recycling pool SVs depends on both their mobilization to the sites of exocytosis and vesicle formation by endocytosis. Evaluation of the FM1-43 uptake showed that propionate does not disturb the endocytosis responsible for SV reformation after exocytosis. However, propionate markedly reduces the rate of FM1-43 dye release from SVs during a 20-Hz stimulation. This directly indicates that the delivery of SVs to the AZ is inhibited. The rate of FM1-43 release was markedly low in frog NEs during the first minute of the 20-Hz stimulation. This fact is consistent with our assumption about an impaired mobilization of the SVs from the recycling pool upon intracellular acidosis.

The mechanisms regulating SV mobilization are organized hierarchically and in a coordinated manner. Cholesterol, its content in membranes, and its metabolites act as potent regulators of SV transport in both the CNS and neuromuscular junctions [22, 28, 29, 30, 31]. Previously, we found that the main cholesterol metabolite in the brain (namely, 24-hydroxycholesterol), which is predominantly produced by neurons (including in synaptic regions), can enhance the involvement of the recycling pool SVs in neurotransmitter release in the mouse neuromuscular junctions [21]. The effect of the hydroxycholesterol depends on protein kinase G, which controls the function of the SV recycling pool in frog NEs [19]. 24-Hydroxycholesterol was found to accelerate the FM1-43 release during exocytosis in mouse and frog NEs, while propionate completely prevents its effect. Therefore, intracellular acidification may be the predominant factor precluding the increased neurotransmitter release in response to humoral stimuli.

The relationship between intracellular acidosis and SV mobilization possibly has a physiological and (or) pathological significance. A transient decrease in the pH in NEs [5, 6, 7] can suppress the mobilization of SVs in order to inhibit neurotransmitter release upon high activity. Thus, a negative feedback loop can form, limiting the release upon intense activity. Inhibition of SV delivery to the AZ can also provide sufficient time for endocytosis of SV to complete and, therefore, replenishment of the recycling pool. Decreased intracellular pH in neurons is observed in patients with a wide range of diseases (metabolic disorders, ischemia, epileptic activity, and neurodegenerative diseases) and in aging [32, 33, 34]. Excess glutamate and acetylcholine release cause damage to the central and neuromuscular synapses [35, 36]. Olesoxime, which can increase the influx of chloride anions (and, therefore, protons as well) into NEs and inhibit neurotransmitter release, exhibits a pronounced neuroprotective effect [31]. Similarly, the antiepileptic drug levetiracetam reduces pH in neocortical neurons, thereby contributing to the anticonvulsant properties of the drug [37]. A slight intracellular acidification may also underlie the anticonvulsant effect of short-chain monocarboxylates and ketone bodies [23]. Systemic ketoacidosis has the potential to affect motor performance by inhibiting neuromuscular transmission at the level of SV mobilization.

The molecular mechanism underlying the effect of intracellular pH on SV translocation to the AZ is unknown. This mechanism can be associated with the changes in mitochondrial function [38], calcium signaling [39], and protein-protein interactions [40]. The fact that the mobilization stage of SVs from the recycling pool is highly sensitive to pH changes suggests that there is a pH sensor. Probably, mobilization of SVs into the recycling pool along actin filaments using motor proteins is suppressed as pH in NEs decreases. So, local interactions between F-actin and myosin strongly depend on pH [41].

## CONCLUSIONS

The pH of the cytoplasm of presynaptic nerve terminals decreases in response to increased synaptic activity under physiological conditions. A more pronounced drop in the pH can occur in disease. In the present study, we obtained data for the first time indicating that intracellular acidification can suppress neurotransmission by inhibiting the synaptic vesicle mobilization in the active zone upon high activity. This phenomenon can be part of a complex mechanism that regulates neurotransmission based on the acid-base processes in neurons.

## Abbreviations

AZ: active zone
EPP: end-plate potential
NE: nerve ending
SV: synaptic vesicle
